# Cholesteatoma Severely Impacts the Integrity and Bone Material Quality of the Incus

**DOI:** 10.1007/s00223-023-01144-6

**Published:** 2023-10-24

**Authors:** Maximilian M. Delsmann, Paul Bonik, Ana Ocokoljic, Sophia M. Häussler, Klaus Püschel, Mark Praetorius, Michael Amling, Jonathan Peichl, Tim Rolvien

**Affiliations:** 1 Division of Orthopaedics, Department of Trauma and Orthopaedic Surgery, University Medical Center Hamburg-Eppendorf, Martinistraße 52, 20246 Hamburg, Germany; 2 Department of Osteology and Biomechanics, University Medical Center Hamburg-Eppendorf, Hamburg, Germany; 3 Department of Otorhinolaryngology, University Medical Center Hamburg-Eppendorf, Hamburg, Germany; 4 Department of Legal Medicine, University Medical Center Hamburg-Eppendorf, Hamburg, Germany

**Keywords:** Bone, Mineralization, Cholesteatoma, Auditory ossicles, Hearing

## Abstract

Cholesteatoma can lead to progressive destruction of the auditory ossicles along with conductive hearing loss but precise data on the microstructural, cellular, and compositional aspects of affected ossicles are not available. Here, we obtained incus specimens from patients who had cholesteatoma with conductive hearing loss. Incudes were evaluated by micro-computed tomography, histomorphometry on undecalcified sections, quantitative backscattered electron imaging, and nanoindentation. Results were compared with two control groups taken from patients with chronic otitis media as well as from skeletally intact donors at autopsy. The porosity of incus specimens was higher in cholesteatoma than in chronic otitis media, along with a higher osteoclast surface per bone surface. Histomorphometric assessment revealed higher osteoid levels and osteocyte numbers in cholesteatoma incudes. Incudes affected by cholesteatoma also showed lower matrix mineralization compared with specimens from healthy controls and chronic otitis media. Furthermore, the modulus-to-hardness ratio was higher in cholesteatoma specimens compared with controls. Taken together, we demonstrated increased porosity along with increased osteoclast indices, impaired matrix mineralization, and altered biomechanical properties as distinct features of the incus in cholesteatoma. Based on our findings, a possible impact of impaired bone quality on conductive hearing loss should be further explored.

## Introduction

Cholesteatoma is a benign, destructive, squamous keratinizing epithelial lesion in the middle ear [1]. With peaks in prevalence among Caucasian populations, the incidence has been estimated at 9.2 per 100,000 adults with a slight predominance in males, making it a common encounter in clinical practice [2]. Likely originating from the lateral epithelium of the tympanic membrane, cholesteatoma forms in the pneumatized aspects of the temporal bone, where it tends to grow into expansive and destructive masses leading to erosion of local bone and soft tissue structures [3]. Resulting complications include conductive hearing loss, osseous destruction of the ossicular chain, and perforation of the tympanic membrane. Other complications such as facial nerve palsy, intracranial abscess, cerebral sinus thrombosis, labyrinthitis, and destruction of inner ear structures may also occur [4, 5].

In acquired cholesteatoma, the ossicular chain is usually among the first damaged structures. If damage is found, the incus is involved in nearly all the cases, presumably due to its size and exposed setting inside the tympanic cavity [2]. Previous studies have reported increased osteoclastic resorption of auditory ossicles, which has been associated with low pH, bacterial colonization, and cytokine expression [6]. The main objective of current research is to identify the factors that drive the rate of cholesteatoma growth and lead to the osseous destruction process. The growth rate displays a high interindividual variance, likely depending on the stage of inflammation, paracrine interaction, and microbial coinfection. Several cytokines have been detected in cholesteatoma tissue and proliferation markers have been linked to progression [7, 8]. Especially increased receptor activator of NFκB ligand (RANKL) expression has been linked to increased bone resorption in cholesteatoma [9]. Lipopolysaccharides, as they naturally occur in Gram-negative bacteria, have been proven to accelerate keratinocyte proliferation and inflammation [6, 10, 11]. Recent findings from a single-cell RNA sequencing analysis of human cholesteatoma specimens suggest that unique activin A-producing fibroblasts are responsible for bone destruction by inducing local osteoclast formation [12].

In deciphering the mechanisms that lead to erosion of the ossicles (malleus, incus, and stapes), it is important to recognize that they differ from other bones in several ways. The extracellular matrix is highly mineralized, with ossicles generally displaying low porosity and a low number of osteocyte lacunae with a high proportion of empty and hypermineralized (micropetrotic) lacunae [13, 14]. This is closely related to the near absence of bone resorption by osteoclasts under normal conditions, accompanied by premature osteocyte apoptosis observed shortly after completion of ossicular ossification [13, 14]. This distinctive pattern may be the result of low mechanical stimuli, while a preserved morphology, hardness, density, and physical capacity for vibration seems beneficial for unaltered conduction of sound throughout life [13, 15–18].

Detailed histopathologic analyses can provide important insights into both pathophysiology and surgical management of cholesteatoma [19]. However, detailed studies on the skeletal integrity of affected ossicles are scarce. In addition to the microstructure and cellular resorption processes, the bone quality of affected ossicles seems to be of particular interest due to their unique function in sound conduction. Therefore, the aim of this study was to perform a high-resolution multiscale characterization of the microstructure and material quality of incus specimens affected by cholesteatoma.

## Methods

### Study Cohort and Specimens

Consecutive incus specimens were obtained during middle ear surgery (tympanoplasty) in patients with chronic otitis media with or without the presence of a cholesteatoma. If cholesteatoma was detected intraoperatively and the incus was found to be eroded, it was removed and subsequently replaced by a prosthesis (primarily or in a second-look operation). In all patients, clinical characteristics were obtained by retrospective chart review. The indication for surgery was made by a senior otolaryngologist. Next to the clinical specimens, a group of *n* = 8 incus specimens from skeletally healthy individuals collected in the context of a previous postmortem study were reanalyzed [13, 20]. This way, a total of 31 individuals were included (control *n* = 8; chronic otitis media *n* = 9, cholesteatoma *n* = 14). The mean age of the cholesteatoma cohort was 33.7 years (ranging from 7.1 years to 64.6 years), and the sex distribution was approximately even, with 8 men (57.1%) and 6 (42.9%) women. The mean age (*p* = 0.65) and sex distribution (*p* = 0.076) did not differ between the three cohorts. Apart from two patients without detectable hearing loss, all cholesteatoma patients presented with hearing loss in the preoperative examination. Namely, pure-tone audiometry revealed the presence of conductive hearing loss in nine (64.3%) and mixed hearing loss in three patients (21.4%).

### Micro-Computed Tomography (μ-CT)

To visualize the incus three-dimensionally and to quantify the porosity, specimens were imaged using a Scanco μCT 42 (Scanco Medical AG, Brüttisellen, Switzerland). The scans were performed at a resolution of 15 μm at 55 kV and 145 μA. The porosity (%) was defined as the fraction of non-osseous volume within the total bone volume as described previously [21].

### Sample Preparation, Histology and Histomorphometry

Specimens were fixed in 3.7% formaldehyde, dehydrated in an ascending ethanol series, and embedded undecalcified in polymethyl methacrylate (PMMA). The embedded samples were cut into 4 µm sections using a rotary microtome (CVT 4060E, microTec, Walldorf, Germany). Staining was performed with von Kossa-van Gieson, trichrome Goldner, and toluidine blue according to previously described protocols [22]. In accordance with ASBMR guidelines [23], histomorphometric analysis was performed using a light microscope (Axioskop 40, Carl Zeiss Vision GmbH, Germany) equipped with Osteomeasure Software (OsteoMetrics Inc., Atlanta, USA).

### Quantitative Backscattered Electron Imaging (qBEI)

The embedded incus specimens were polished to a coplanar surface, carbon-coated, and analyzed using a scanning electron microscope (LEO 435 VP, LEO Electron Microscopy Ltd.; Cambridge, UK) with a backscattered electron detector (Type 202; K.E. Developments Ltd.; Cambridge, UK). Quantitative backscattered electron imaging (qBEI) was performed to determine the bone mineral density distribution (BMDD) and osteocyte lacunar characteristics according to standard procedures at 20 kV and 680 pA at a constant working distance [24, 25]. The generated gray values correlate with the mean calcium content (mean Ca-Wt%) of the cross-sectioned bone [26]. Brightness and contrast of the qBEI images were calibrated with carbon (gray value: 4.8) and aluminum standards (gray value: 222). The qBEI images were acquired at 120 × magnification, before being analyzed using ImageJ (ImageJ 1.42, National Institutes of Health, Bethesda, USA) [27] and a custom MATLAB-based script (TheMathWorks, Inc., Natick, USA).

### Nanoindentation

Nanoindentation was performed using an iMicro nanoindenter (KLA instruments, CA, USA) equipped with a Berkovich diamond tip. The dehydrated, PMMA-embedded specimens were polished and mounted on a platform. With a Poisson’s ratio of 0.3, the surface was approached at a speed of 100 nm/s, while the depth limit was set at 3000 nm and the strain target rate was set at 0.05 1/s. Thirty indents were placed in a peripheral bone region. All valid indents per ossicle were averaged to calculate the mean hardness and the Young’s modulus (GPa). Furthermore, the modulus/hardness ratio, a surrogate measure for fracture toughness, was calculated [28].

### Statistical Analysis

GraphPad Prism software (version 9.0, GraphPad Software, La Jolla, USA) was used for statistical analysis. Continuous variables are given as absolute values or as mean ± standard deviation (SD). To evaluate the normal distribution of the data, the Shapiro–Wilk test was used. One-way ANOVA with Tukey’s multiple comparison test was used to compare normally distributed data between three groups, while the Kruskal–Wallis test with Dunn's multiple comparison test was performed for nonparametric data. To compare two groups, the Student's *t* test was used for normally distributed data and the Mann–Whitney *U* test was used for nonparametric data. The level of significance was defined as *p* < 0.05.

## Results

### Incus Porosity in Cholesteatoma is Accompanied by Increased Osteoclast Indices

**Fig. 1:**
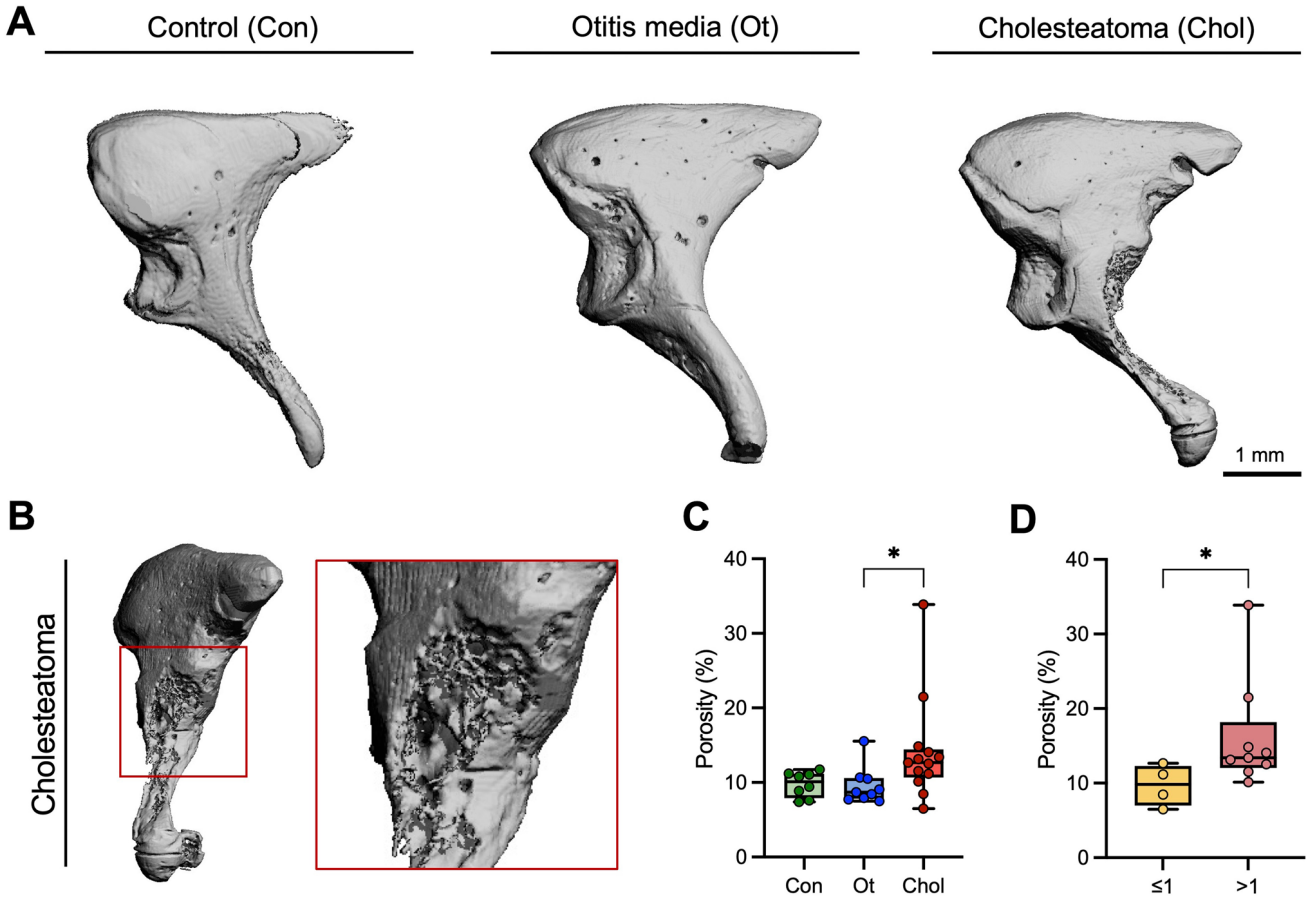
Eroded surfaces and increased porosity characterize the incus of cholesteatoma patients. **A** Representative three-dimensional μCT reconstructions of the incus in control (left panel), chronic otitis media (middle panel) and cholesteatoma patients (right panel). **B** Higher magnification of the resorption zone (eroded surface) of a cholesteatoma incus, lateral view with corresponding magnification (red box). **C** Evaluation and comparison of the porosity between the groups. Kruskal–Wallis test with Dunn's multiple comparison test. **D** Comparison of the porosity according to the number of macroscopically affected ossicles. Mann–Whitney *U* test. *p < 0.05

**Fig. 2:**
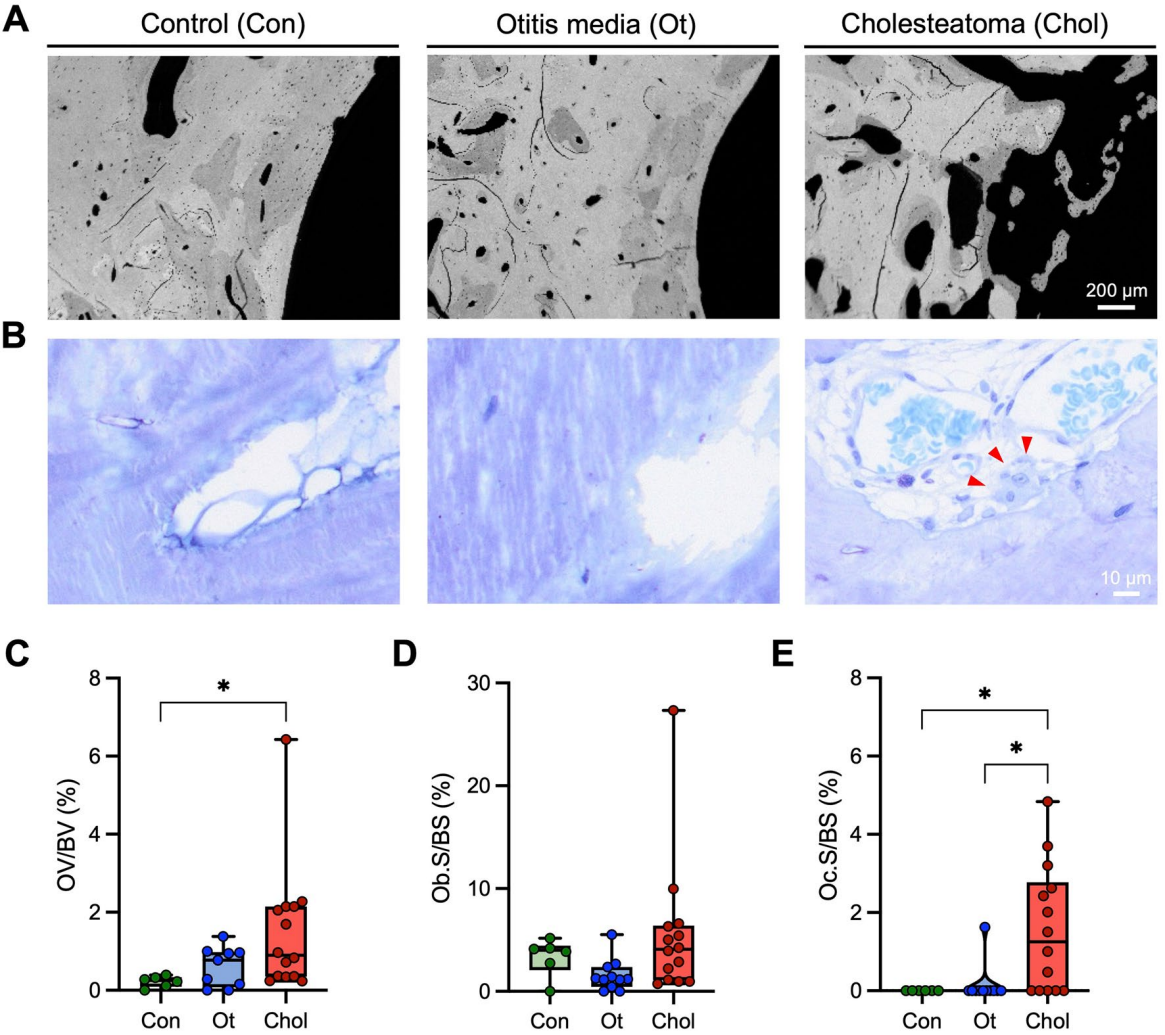
Increased osteoid indices and abundance of osteoclasts in cholesteatoma. **A** Overview images of the incus obtained by backscattered electron microscopy. **B** Representative histological images of toluidine blue stained sections. Red arrowheads indicate a multinucleated osteoclast. **C** Quantification of the osteoid volume per bone volume (OV/BV), **D** osteoblast surface per bone surface (Ob.S/BS), and **E** osteoclast surface per bone surface (Oc.S/BS). Kruskal–Wallis test with Dunn's multiple comparison test was performed in panels **C**–**E**. *p < 0.05

Microstructural analysis of the incus by µ-CT revealed resorption zones (i.e., porosity) in a subset of cholesteatoma specimens (Fig. 1 A, B), which were not detected in any of the specimens from the other two groups. Accordingly, the mean porosity was higher in cholesteatoma (14.1 ± 6.9%) compared to chronic otitis media (9.6 ± 2.5%; *p* = 0.036) (Fig. 1 C). A subgroup analysis showed that the incus porosity was higher in patients with intraoperative macroscopic affection of more than one ossicle (Fig. 1 D). Backscattered electron microscopy and undecalcified histologic sections confirmed the porosity and the eroded surfaces with visible osteoclasts, respectively (Fig. 2 A, B). Histomorphometry revealed a higher osteoid volume per bone volume (OV/BV) in the incus of cholesteatoma patients (1.6 ± 2.2%) compared to controls (0.2 ± 0.1%; *p* = 0.037; Fig. 2 C). However, there was no difference in OV/BV between cholesteatoma and chronic otitis media. While the osteoblast surface per bone surface (Ob.S/BS) was similar in all groups (Fig. 2 D), the osteoclast surface per bone surface (Oc.S/BS) was higher in cholesteatoma (1.56 ± 1.61%) compared to chronic otitis media (0.15 ± 0.49%, *p* = 0.012) and control (0.0 ± 0.0%, *p* = 0.019) incudes (Fig. 2 E).

**Fig. 3:**
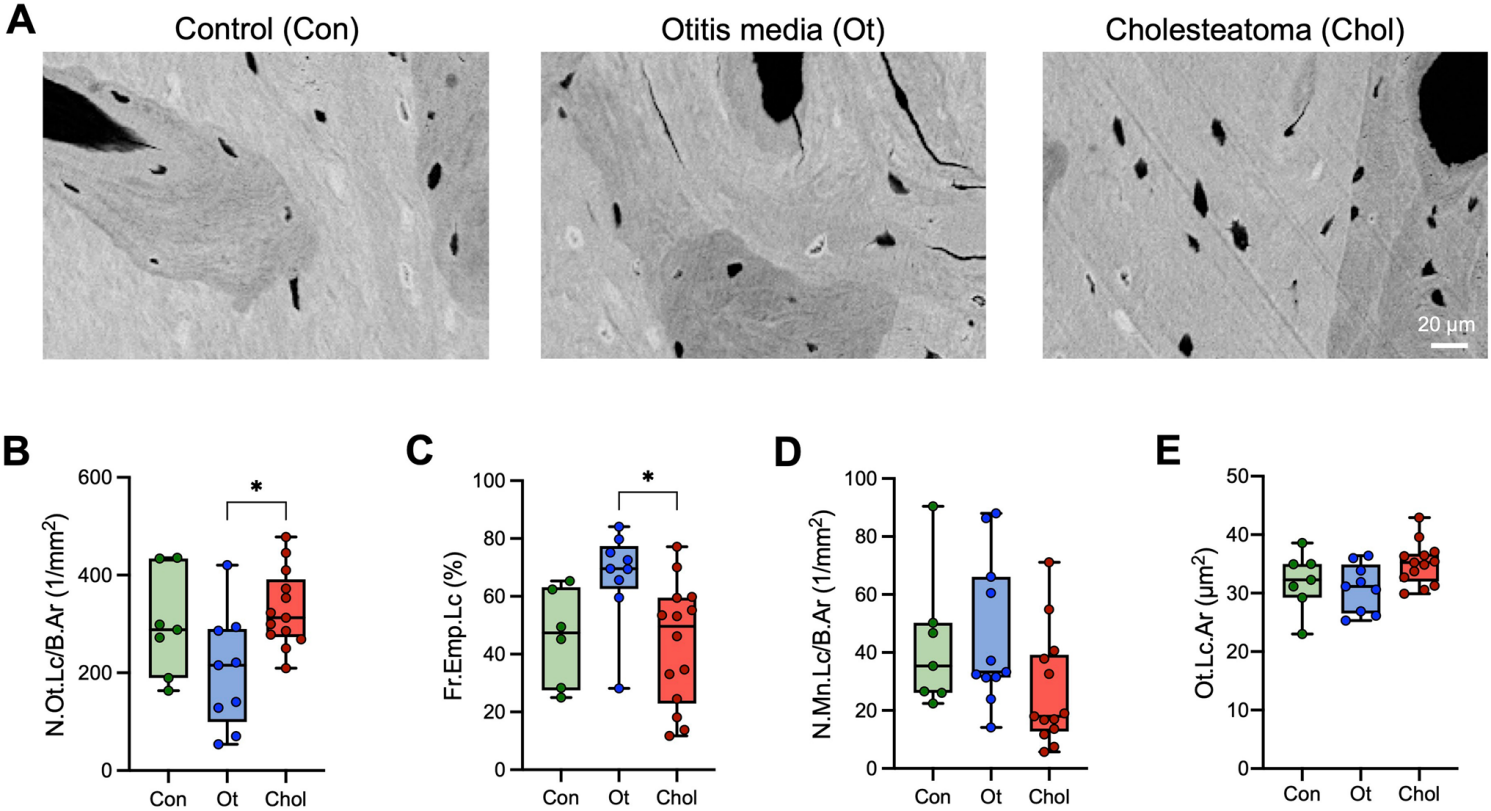
Increased osteocyte lacunar numbers with signs of higher viability in cholesteatoma. **A** Representative high magnification qBEI images showing an overall high frequency of mineralized lacunae. **B** Quantification of the number of the osteocyte lacunae (N.Ot.Lc/B.Ar), **C** fraction of empty lacunae per total number of lacunae (Fr.Emp.Lc), **D** number of mineralized lacunae per bone area (N.Mn.Lc/B.Ar), and **E** osteocyte lacunar area (Ot.Lc.Ar). ANOVA with Tukey’s multiple comparison tests were performed in panels **B**–**E**. *p < 0.05

Analysis of osteocyte properties (Fig. 3 A) revealed a higher number of total osteocyte lacunae (N.Ot.Lc/B.Ar) in cholesteatoma (329.7 ± 78.8 1/mm^2^) compared to chronic otitis media (203.4 ± 118.4 1/mm^2^; *p* = 0.018) (Fig. 3 B) as well as a lower fraction of empty lacunae (Fr.Emp.Lc, 43.6 ± 21.1% *vs.* 67.1 ± 16.3%; *p* = 0.016 (Fig. 3 C). However, no differences between the groups could be detected in the number of mineralized lacunae per bone area (N.Mn.Lc/B.Ar) or the osteocyte lacunar area (Ot.Lc.Ar) (Fig. 3 D, E).

### Matrix Hypomineralization as a Distinct Phenomenon in Cholesteatoma

**Fig. 4:**
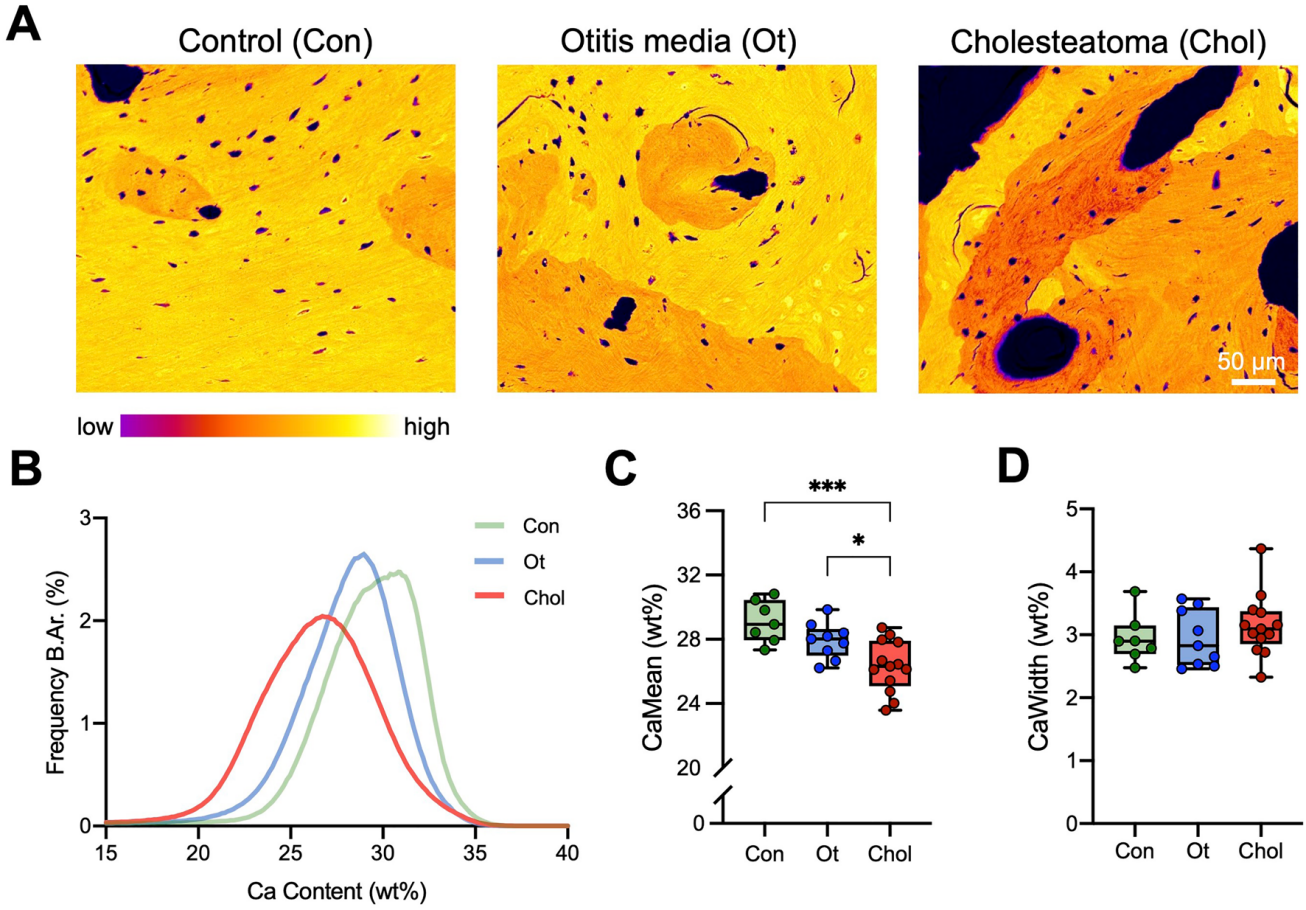
Lower matrix mineralization in incudes obtained from cholesteatoma compared to control and otitis media patients. **A** Representative, pseudocolored qBEI images of the incus in controls, chronic otitis media and cholesteatoma. **B** BMDD histograms of the incus of control (green curve), chronic otitis media (blue curve), and cholesteatoma patients (red curve). **C** Quantification of the mean calcium content (CaMean). **D** Quantification of the mineralization heterogeneity (CaWidth). ANOVA with Tukey’s multiple comparison tests were performed in panels **C** and **D**. *p < 0.05, ***p < 0.001

High-resolution imaging of the incus by qBEI indicated an impaired bone mineralization of cholesteatoma incudes (Fig. 4 A). BMDD histograms indicated a leftward shift with a wider calcium distribution curve in cholesteatoma compared to both chronic otitis media and controls (Fig. 4 B). The mean calcium content (CaMean) was lower in cholesteatoma (26.3 ± 1.6Wt%) compared to chronic otitis media (27.9 ± 1.Wt%; *p* = 0.0376) and control incudes (29.1 ± 1.3Wt%; *p* = 0.0007) (Fig. 4 C). However, no differences in the heterogeneity of the mineralization (CaWidth) could be detected between the groups (Fig. 4 D).

### Differences in Mineralization are Paralleled by Increased Modulus/Hardness Ratio

**Fig. 5:**
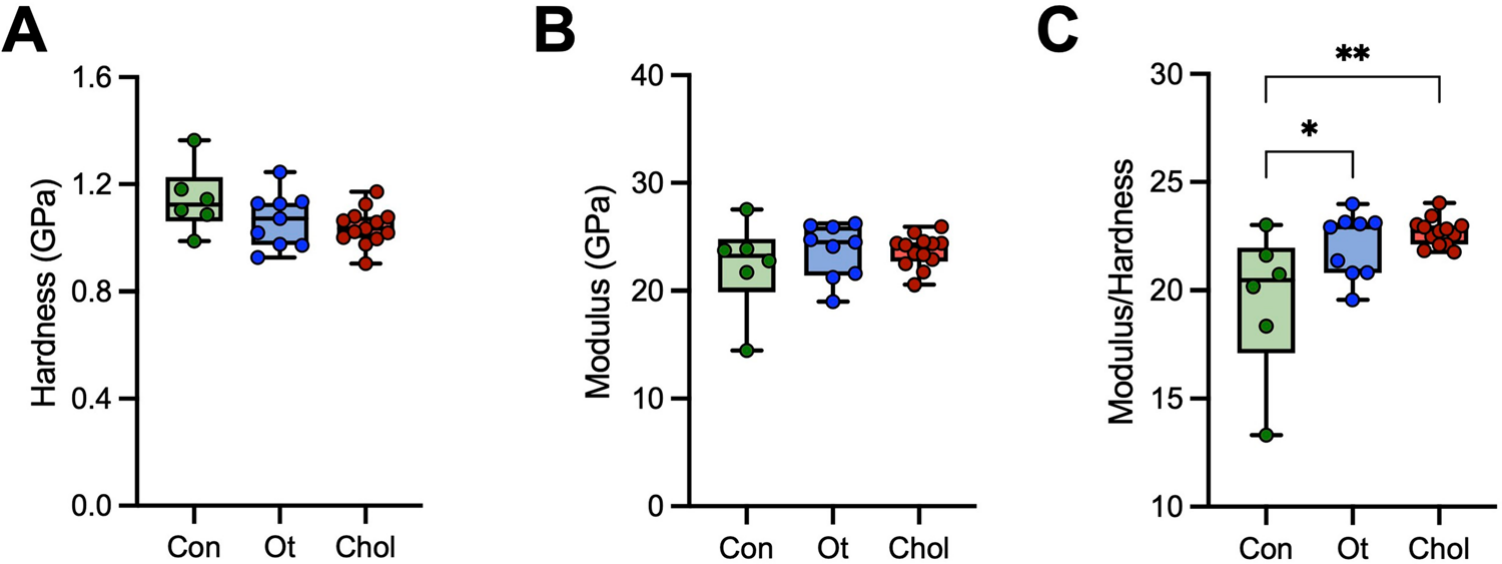
Increased modulus/hardness ratio as a biomechanical feature of incudes affected by cholesteatoma. **A** Quantification of hardness, **B** modulus, and **C** modulus/hardness ratio. ANOVA with Tukey’s multiple comparison tests were performed in all panels. *p < 0.05 **p < 0.005

Nanoindentation was used to study the biomechanical properties of the ossicles. Although tissue hardness showed a similar trend to the CaMean values from the qBEI analysis, both hardness (Fig. 5 A) and Young’s modulus (Fig. 5 B) showed no significant differences between the study groups. However, the modulus/hardness ratio was higher both in cholesteatoma (22.68 ± 0.64; *p* = 0.0048) and chronic otitis media (22.08 ± 1.48; *p* = 0.0252) incudes compared to controls (19.53 ± 3.41) (Fig. 5 C). No differences could be detected regarding the comparison of the modulus/hardness ratio between the cholesteatoma and chronic otitis media specimens.

## Discussion

In this study, we performed a comprehensive investigation of incus specimens obtained from cholesteatoma patients, focusing on microstructural, cellular, and compositional alterations. To gain insights into the osseous destruction processes, we took advantage of an ex situ, multiscale imaging approach, comparing the findings with incus specimens from a clinical cohort of patients with otitis media without cholesteatoma and a postmortem cohort of skeletally intact controls. Resorption zones and porosity were found in a subset of cholesteatoma incudes, along with increased osteoclast indices compared to controls and chronic otitis media. In addition, the incus of cholesteatoma patients showed a lower degree of matrix mineralization, higher osteoid volume, and higher osteocyte numbers. Notably, the number of ossicles macroscopically affected by bone destruction, based on intraoperative assessments, appeared to be associated with the porosity of the incus, presumably reflecting the progression of the disease.

Theoretically, the inflammatory environment in cholesteatoma causes porosity by activating bone resorption. Increased porosity and osteoclastic bone resorption were detected to varying degrees in cholesteatoma incudes studied here. Previous studies confirmed that the resorptive process in cholesteatoma is driven by activated osteoclasts. In a scanning electron microscopy study on the morphometry of erosions in auditory ossicles affected by cholesteatoma, osteoclastic resorptive lacunae appeared similar to those in osteoporotic femoral necks [29]. Furthermore, osteoclasts have been shown to be activated by paracrine secretion of RANKL by fibroblasts and lymphocytes located in the peri-matrix and triggered by inflammation [9, 30, 31]. Whereas Imai and colleagues demonstrated a significantly higher number of osteoclasts in their study [30], Koizumi et al. failed to find osteoclasts on affected bone structures [32]. These divergent results could be due to possible stage-specific effects and the fact that osteoclasts are characterized by a transient presence and short life span (1–25 days) [33]. Overall, our data reveal a heterogeneous pattern of resorption-associated porosity in cholesteatoma.

In addition to the increased porosity, which could be quantitatively measured and compared with adequate control groups for the first time, the detected matrix hypomineralization by qBEI was the second important result of our study. In a previous study, inflammatory incudes also showed lower mineralization, although this quantification was limited to bone mineral density via µ-CT [34]. Since hypomineralization of auditory ossicles has been associated with conductive hearing loss in the context of genetic bone diseases [18], it may also contribute to conductive hearing loss in cholesteatoma. The mechanisms leading to ossicular hypomineralization in cholesteatoma remain unclear, although our data suggest a possible link to higher bone remodeling, consistent with the higher osteocyte numbers observed. It is interesting to note that the trend of higher numbers of osteocyte lacunae with decreased rates of empty lacunae was not fully recapitulated by decreased numbers of mineralized lacunae. As a high number of mineralized osteocyte lacunae has been previously reported as a sign of overall low bone remodeling and premature aging of the bone matrix in auditory ossicles [13], our results suggest that this condition can be partially reversed in the case of the cholesteatoma.

In almost all affected patients, we detected conductive hearing loss, which is generally caused by ossicular damage, loss of continuity of the ossicular chain, but also due to the mere presence of cholesteatoma matrix in the middle ear [35]. In this context, an association between cholesteatoma-related ossicular erosions and conductive hearing loss has been demonstrated in 158 patients with chronic otitis media [35]. While we were not able to investigate such associations in detail in our study due to the high frequency of affected patients with conductive hearing loss, we found an association between the number of affected ossicles according to intraoperative findings and incus porosity. This suggests that the spread of the chronic inflammatory process may influence the extent of resorption. Interestingly, we also found an increased modulus-to-hardness ratio in cholesteatoma incudes using nanoindentation. This ratio was reported as a surrogate marker for fracture toughness, suggesting improved mechanical competence. However, as the function of ossicles in the context of sound conduction in the middle ear is fundamentally different from other bones, an increased modulus-to-hardness ratio, indicating higher elasticity, could also portend impaired sound conduction due to altered ossicular vibrational capacity.

As there is no pharmacological treatment for cholesteatoma available today, the standard procedure mainly relies on the surgical removal of the affected tissue with subsequent reconstruction of potentially damaged structures such as the tympanic membrane and ossicular chain (tympanoplasty). With a more profound understanding of the molecular patterns and overall mechanisms that drive the pathogenesis and progression of cholesteatoma, pharmaceutical intervention may become a valid alternative to surgery in the early stages and prophylaxis for the high rate of recurrence after surgery. In preventing bone destruction, medications commonly used in the treatment of osteoporosis may be a possible treatment option, possibly through topical application. Evidence from an in vitro study with cultured keratinocytes from cholesteatoma showed that the bisphosphate pamidronate inhibited bone resorption [36]. As the resorptive process appears to be driven by osteoclasts activated by RANKL, bisphosphonates or recombinant monoclonal RANKL antibodies might prevent complications and serve as a bridge to surgery. Moreover, since a high dose calcium diet prevented hypomineralization of auditory ossicles in vitamin D receptor deficient mice [37], modulation of calcium homeostasis might also be effective as an additive measure to improve bone mineralization of ossicles in cholesteatoma.

In conclusion, we demonstrated resorption-related porosity, matrix hypomineralization and altered biomechanical properties as distinct phenomena of the incus in cholesteatoma patients. These results indicate that the low bone turnover state normally present in auditory ossicles can be activated under certain conditions, which calls for further mechanistic investigation. The possible dependence of conductive hearing loss on ossicular quality in cholesteatoma as well as other ear and bone diseases should be further investigated.

## Data Availability

The datasets used and/or analyzed during the current study are available from the corresponding author on reasonable request.
